# Finger Fractures as an Early Manifestation of Primary Hyperparathyroidism Among Young Patients: A Case Report of a 30-Year-Old Male With Recurrent Osteoporotic Fractures

**DOI:** 10.1097/MD.0000000000003683

**Published:** 2016-05-20

**Authors:** Akihiko Ozaki, Tetsuya Tanimoto, Eiki Yamagishi, Shunsuke Sato, Manabu Tsukada, Toyoaki Sawano, Claire Leppold, Kenji Tsuda, Takanori Asakura, Masaharu Tsubokura, Shigeaki Kato, Masahiro Kami, Hiromichi Ohira

**Affiliations:** From the Department of Surgery, Minamisoma Municipal General Hospital, Minamisoma (AO, MT, TS, HO); Department of Internal Medicine, Jyoban Hospital of Tokiwakai Group, Iwaki (TT); Department of Orthopaedic Surgery, Iwase General Hospital, Sukagawa (EY); Department of Orthopaedic Surgery (SS); Department of Research, Minamisoma Municipal General Hospital, Minamisoma, Fukushima (CL); Department of Hematology and Reumatology, Teikyo University Chiba Medical Center, Ichihara, Chiba, Japan (KT); Division of Pulmonary Medicine, Department of Medicine, Keio University School of Medicine, Tokyo (TA); Department of Radiation Protection, Minamisoma Municipal General Hospital, Minamisoma (MT); Research Institute of Innovative Medicine, Jyoban Hospital, Iwaki, Fukushima (SK); and Medical Governance Research Institute, Tokyo (MK), Japan.

## Abstract

Osteoporosis and osteoporotic fractures represent a substantial health burden, and predominantly affect the elderly. Younger generations may also develop these conditions because of various predisposing conditions, including primary hyperparathyroidism. However, little information is available regarding early skeletal manifestations of primary hyperparathyroidism.

A 30-year-old Japanese male presented with pain in his left wrist, and was diagnosed with a distal radius fracture. During surgery, we noticed decreased bone strength of the fracture site. Further investigation found osteoporosis and primary hyperparathyroidism owing to a solitary parathyroid adenoma, which was resected without significant complications. History revealed that the patient suffered a metacarpal bone fracture of his right fifth bone 6 months earlier. Although serial x-rays at that time had shown rapidly developed cortical bone erosion around the fractured finger, the possibility of primary hyperparathyroidism was overlooked because of poor awareness of the condition, leading to a 6-month delay in the diagnosis of primary hyperparathyroidism.

Clinicians should be aware that finger fractures may be an early skeletal manifestation of primary hyperparathyroidism that can help achieve a prompt diagnosis of the condition, especially when they occur in young adults in the absence of major trauma.

## INTRODUCTION

Osteoporosis and osteoporosis-related fragility fractures are a significant health burden among the elderly worldwide.^1,2^ These fractures occur most frequently in the spine, proximal humerus, proximal femur, and distal radius (referred to as major osteoporotic fractures), possibly contributing to chronic pain, physical and psychosocial disability, and a worsened mortality^1–3^

Although young adults experience bone fractures mainly because of major traumas,^1^ osteoporotic fractures may also occur because of medication use or various underlying diseases.^3^ Primary hyperparathyroidism (PHPT) is associated with increased osteoclast activity and bone turnover particularly in cortical bones,^4^ and is a predisposing condition, which can lead to osteoporosis and related fractures in younger generations.^5^ A recent study suggests that the prevalence of PHPT in the general population is 1 to 4 in 1000, with females 2 times more likely to be affected than males, and the majority of patients diagnosed in their 50s or 60s.^5^ Currently, 70% to 80% of patients with PHPT in developed countries have the condition incidentally discovered because of hypercalcemia,^5^ and this proportion has been consistently rising.^6^ However, young individuals in their 20s or 30s often receive no health checkups, including blood analysis.^7^ Moreover, testing for osteoporosis is not generally recommended when males younger than 50 years or premenopausal females fracture a bone.^3^ Considering the relatively low prevalence of PHPT in this population^5^ and limited clinician awareness, there is a large potential for PHPT-induced osteoporosis to be missed in young adults with fractures, resulting in delays in diagnosis and treatment. These delays may be risky, as PHPT patients can develop significant fragility fractures such as femoral fractures in the absence of major trauma.^8,9^ It is therefore imperative to recognize early skeletal symptoms of PHPT in young adults, and to make a prompt and accurate diagnosis. However, this is made difficult by the lack of general information concerning skeletal manifestations indicative of early-stage PHPT.

We experienced the case of a young male with a delayed diagnosis of PHPT. Although he fractured a metacarpal bone of right fifth finger half a year before the PHPT diagnosis, involvement of PHPT was overlooked at the time.

## CASE PRESENTATION

**FIGURE 1:**
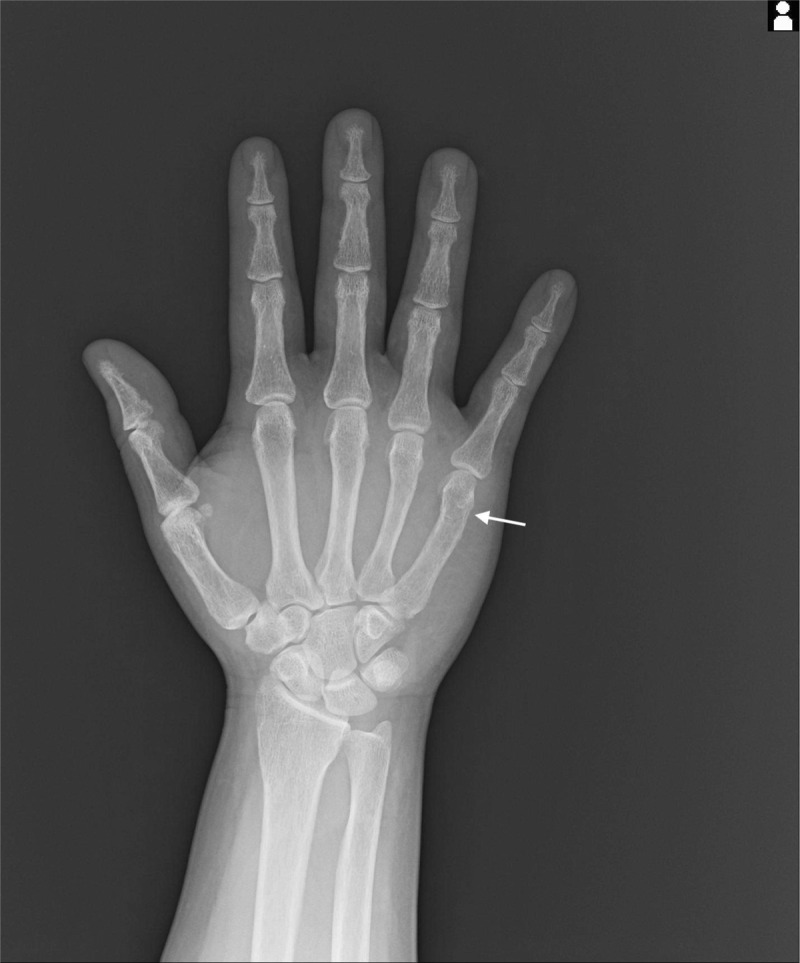
An x-ray showing the metacarpal bone fracture at right fifth bone of the patient on the day of the injury (arrow).

**FIGURE 2:**
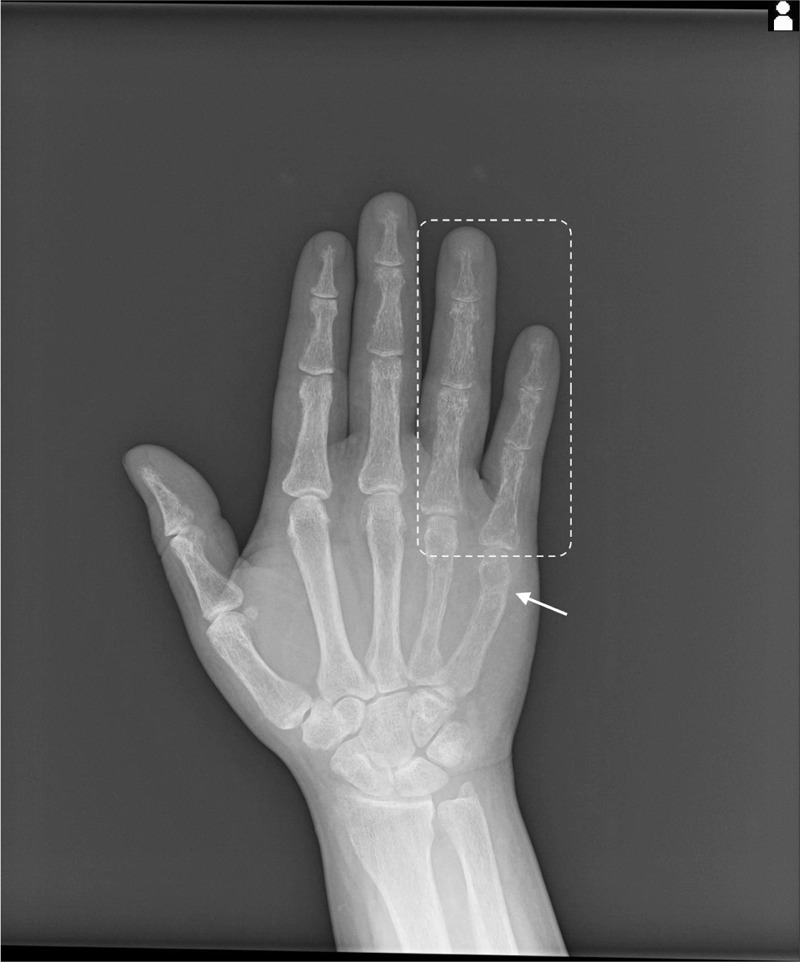
An x-ray showing the fracture (arrow) and cortex bone erosion of right fourth and fifth finger (surrounded area) 2 weeks following the injury.

A 30-year-old Japanese male presented to our orthopedic department with left wrist pain following a low-energy simple fall. He has not received health checkups for several years, and his family history was unremarkable. Six months before, the patient had lightly hit his right hand against the wall, resulting in a metacarpal bone fracture of his right fifth finger. At that time, x-ray imaging showed no remarkable findings other than the fracture (Figure 1), and conservative treatment with external fixation of the site was selected. However, a follow-up x-ray 2 weeks later revealed substantial cortical bone erosion of his right fourth and fifth fingers under fixation (Figure 2). Although the attending doctor recognized that this was an unusual finding, he judged that a reduction of mechanical stress had caused a decreased bone mineral density (BMD) in the affected fingers, and did not consider the involvement of any predisposing conditions. No further investigation was undertaken. The fracture recovered without sequela.

**FIGURE 3:**
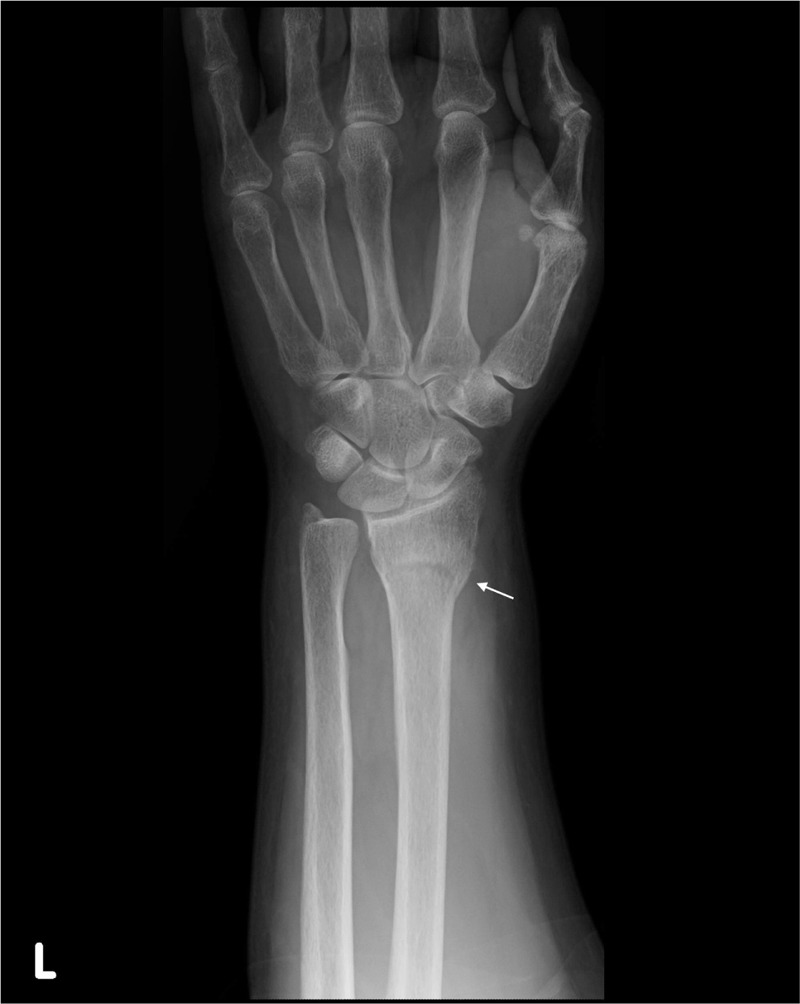
An x-ray showing left distal radius fracture of the patient (arrow).

At the present visit, 6 months following the metacarpal bone fracture diagnosis, the patient was experiencing swelling and tenderness at his right wrist. An x-ray revealed a left distal radius fracture (Figure 3). Internal fixation of the radius was performed, and a decreased strength of the cortical bone at the site was observed by a surgeon during the procedure. After the surgery, thorough examination for osteoporosis was conducted, as follows.

**FIGURE 4:**
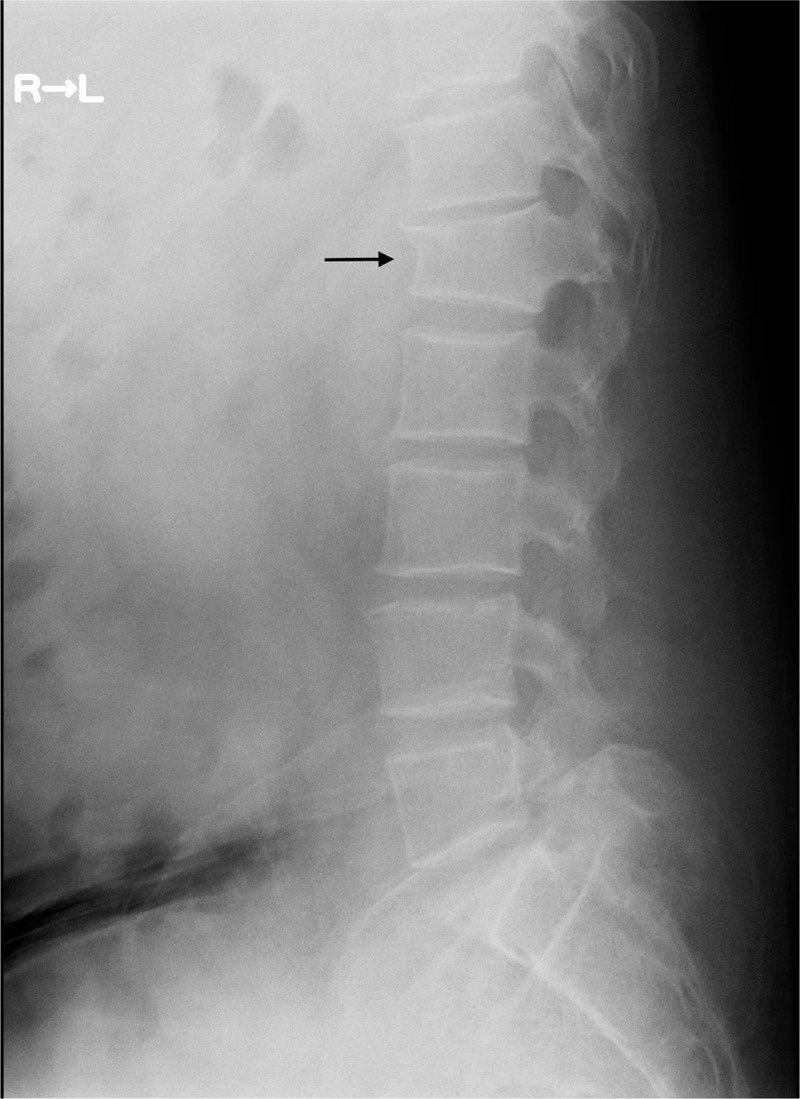
An x-ray showing L-1 compression fracture of the patient (arrow).

**FIGURE 5:**
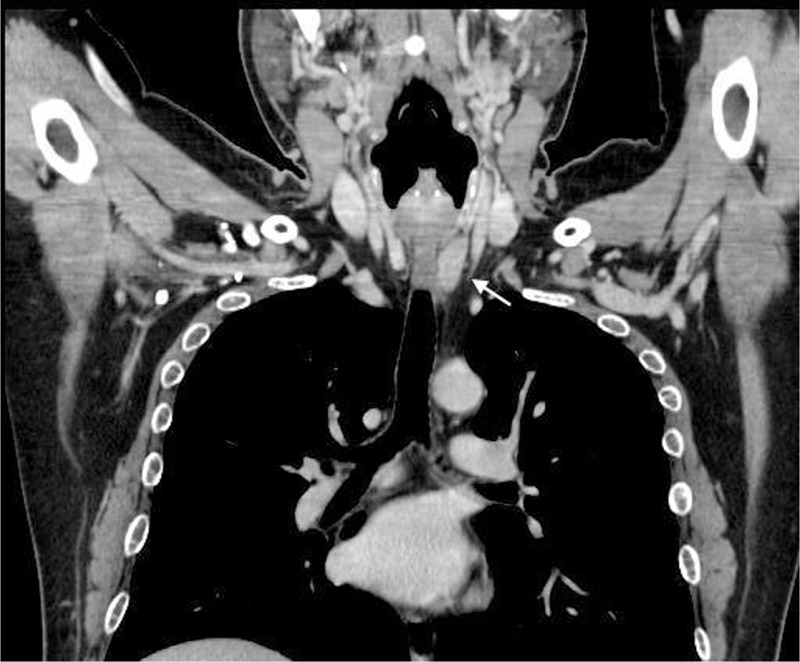
A computed tomography scan showing his parathyroid adenoma (arrow).

**FIGURE 6:**
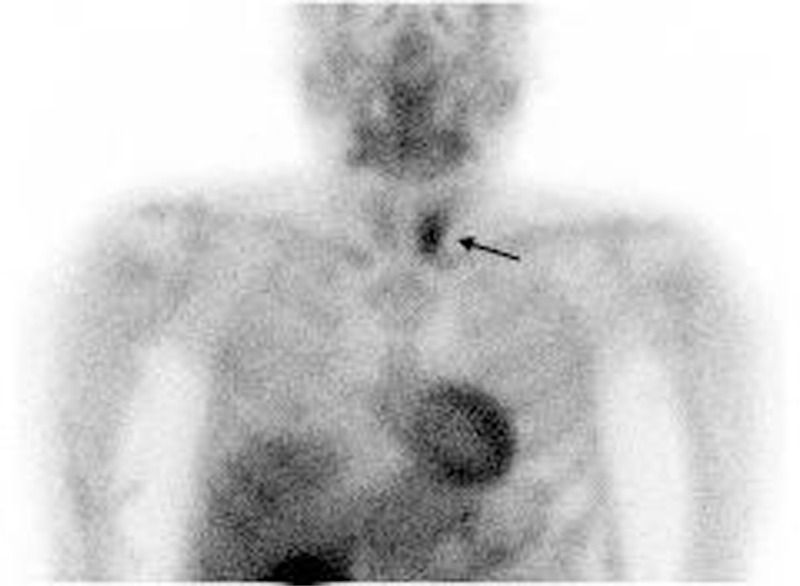
A scintigraphy showing his parathyroid adenoma (arrow).

A second look at the x-ray (Figure 3) indicated a thinning of cortical bone around the fractured radius. Dual-energy x-ray absorptiometry (DXA) revealed osteoporosis, with a femoral neck *z* score −2.7 (normal range: >−2.0) and a lumber spine *z* score −3.7 (normal range: >−2.0). An x-ray of his spine additionally showed an asymptomatic L-1 compression fracture (Figure 4). His serum calcium level and serum parathyroid hormone level were increased at 12.4 mg/dL (normal range: 8.4–10.2), and >3200 ng/L (normal range: 160–520), respectively. Extensive imaging studies including computed tomography (Figure 5) and scintigraphy (Figure 6) showed a parathyroid tumor, adjacent to the inferior margin of the left thyroid lobe, without a finding suggestive of multiple endocrine neoplasia. He was clinically diagnosed with PHPT because of a solitary parathyroid adenoma, and was referred to our surgery department. Excision of his parathyroid tumor was performed, and a subsequent pathological examination confirmed the diagnosis of parathyroid adenoma. The patient was discharged without significant complications.

## DISCUSSION

This case highlights the difficulty in making an early diagnosis of PHPT, particularly among young patients. When young adults develop finger fractures following minimal trauma, clinicians should consider preexisting risk factors for osteoporosis, especially PHPT. In current clinical practice, DXAs of a spine and a femoral bone are established examination methods for confirming a diagnosis of osteoporosis among the elderly.^3,10^ With the exception of vertebral compression fractures and deformities, a plain radiograph of bones only provides limited information to accurately estimate BMD, bone strength, or involvement of osteoporosis.^3,10,11^ In fact, none of the orthopedic specialists we consulted recognized the effect of osteoporosis in the x-ray of the fractured finger at first presentation (Figure 1), before we informed them of the diagnosis. Previous studies have shown that fractures of fingers occur mainly in healthy young males,^12^ and are not generally associated with osteoporosis.^3,12^ However, considering that bone erosion of finger bones can be observed in the early phase of PHPT,^6,13^ it may be reasonable to assume that finger fractures can be an early skeletal manifestation of PHPT. Currently, PHPT in younger generations is usually diagnosed only after the development of serious femoral fractures or multiple fractures, unless incidentally discovered in health checkups.^14^ However, an early diagnosis of PHPT at the time of minor fractures can help to prevent further morbidities. There is a need for awareness among clinicians that fragility fractures may occur at fingers, and other major osteoporotic fracture sites, owing to PHPT in young adults.^15^

Osteoporosis induced by PHPT may rapidly deteriorate during delays in diagnosis, and lead to devastating consequences. In our patient, significant bone erosion at the fixed fingers, a typical skeletal manifestation of PHPT,^6^ had developed within only 2 weeks. This is an extremely atypical finding for a healthy adult, considering that it conventionally takes 2 months before bone loss related to immobilization becomes recognizable on an x-ray.^16,17^ Although there is little information available concerning how fast skeletal manifestations of PHPT develop, PHPT impacts BMD promptly with a 50% average increase of bone turnover compared with healthy populations, and can raise the risk of further fractures without timely intervention,^4,6^ as accentuated in this episode. Half a year following the initial finger fracture of our patient, he experienced a fracture of his right wrist, which is a frequent fracture site induced by PHPT.^15^ Furthermore, it is to be noted that an asymptomatic vertebral compression fracture with unknown onset was detected in our patient. This is another conventional manifestation of PHPT,^15^ which can lead to further complications, including height loss, functional impairment, persistent pain, subsequent fractures, and an increased mortality.^11^ Approximately, 85% of PHPT is caused by a solitary parathyroid adenoma,^5^ and definitive treatment with parathydectomy can improve the BMD of various bones and decrease risk of future fractures.^4,18^ Because of the health-improving measures that can be taken upon a timely diagnosis of PHPT, we underscore the need for clinicians to pay attention to findings suggestive of early-stage PHPT in young adults, such as the finger fractures in the present case.

## CONCLUSIONS

This is a case of young male with multiple osteoporotic fractures owing to a delayed diagnosis of PHPT. This case underlines the importance of considering the potential for PHPT when young adults develop finger fractures in the absence of major trauma. Clinicians should be aware that early management of PHPT is important to prevent devastating consequences, and that finger fractures in young adults can be a very early manifestation of PHPT.
